# Non-congenital severe ocular complications of Zika virus infection

**DOI:** 10.1099/jmmcr.0.005152

**Published:** 2018-05-14

**Authors:** Mussaret B. Zaidi, C. Gustavo De Moraes, Michele Petitto, Juan B. Yepez, Anavaj Sakuntabhai, Etienne Simon-Loriere, Matthieu Prot, Claude Ruffie, Susan S. Kim, Rando Allikmets, Joseph D. Terwilliger, Joseph H. Lee, Gladys E. Maestre

**Affiliations:** ^1^​ Infectious Diseases Research Laboratory, Hospital General O'Horan, Merida, Mexico; ^2^​ Department of Epidemiology and Biostatistics, Michigan State University, East Lansing, MI, USA; ^3^​ Department of Ophthalmology, Columbia University Medical Center, New York, NY, USA; ^4^​ Clinica de Ojos de Maracaibo, Maracaibo, Venezuela; ^5^​ Department of Ophthalmology, King Khaled Eye Specialist Hospital, Riyadh, Saudi Arabia; ^6^​ Functional Genetics of Infectious Diseases Unit, Institut Pasteur, Paris, France, CNRS, UMR2000, Paris, France; ^7^​ Viral Genomics and Vaccination Unit, Institut Pasteur, Paris, France, CNRS, UMR 3965, Paris, France; ^8^​ In-patient Diabetes Unit, St Peter’s Hospital, Albany, NY, USA; ^9^​ Departments of Psychiatry and Genetics and Development, Columbia University Medical Center, New York, NY, USA; ^10^​ Sergievsky Center, Columbia University Medical Center, New York, NY, USA; ^11^​ Division of Medical Genetics, New York State Psychiatric Institute, New York, NY, USA; ^12^​ Public Health Genomics Unit, National Institute for Health and Welfare, Helsinki, Finland; ^13^​ Taub Institute and Department of Epidemiology, Columbia University Medical Center, New York, NY, USA; ^14^​ Laboratory of Neuroscience, University of Zulia, Maracaibo, Venezuela; ^15^​ Department of Biomedical Sciences, Division of Neurosciences, University of Texas Rio Grande Valley School of Medicine, Brownsville, TX, USA; ^16^​ Department of Human Genetics, University of Texas Rio Grande Valley School of Medicine, Brownsville, TX, USA

**Keywords:** Zika virus, dengue, Arbovirus, *Flavivirus*, uveitis, optic neuritis, vision loss, steroid treatment

## Case summary

In 2016, during a major Zika virus (ZIKV) outbreak in Maracaibo, Venezuela, a 49-year-old woman and an unrelated 4-year-old boy developed bilateral optic neuritis 2–3 weeks after presenting an acute febrile illness characterized by low-grade fever, rash and myalgia [1]. Both patients presented sudden, painless bilateral loss of vision with no corneal or uveal abnormalities. Fundoscopic examination revealed oedema of the optic nerve and optic disc pallor. Optical coherence tomography confirmed bilateral optic nerve head swelling in the case of the adult, but it was not carried out in the child. Automated perimetry performed in the adult revealed bilateral diffuse visual field loss. Magnetic resonance imaging of the brain in both cases was unremarkable. Both patients were diagnosed with bilateral optic neuritis of possible infectious or parainfectious origin. Differential diagnoses that were considered and subsequently discarded included arteritic and non-arteritic ischaemic optic neuropathy, and brain disorders such as multiple sclerosis and brain tumours. Both patients were seropositive for anti-ZIKV IgG and seronegative for anti-ZIKV IgM. In addition, both patients were positive for anti-dengue virus (DENV) IgG for all four DENV serotypes. Management included intravenous methylprednisolone for 3 days, followed by oral prednisolone for 11 days. Although the patients presented a modest improvement in their vision, they continued to have visual impairment after several months of follow-up [1].

**Question**

Which of the following statements is accurate about non-congenital severe ocular complications of Zika virus (ZIKV) infection?

**Answer options**

1. They are unique to ZIKV infection and readily distinguishable from complications caused by other flaviviruses.

2. Serious ocular complications are related to the severity of the acute exanthematous illness.

3. The diagnosis can be conclusively established by detecting anti-Zika IgM and/or IgG in the patient’s serum.

4. These complications can lead to permanent visual impairment.

5. There is specific treatment for ocular manifestations caused by ZIKV infection.

## Discussion

**Correct Answer**: 4. These complications can lead to permanent visual impairment.

ZIKV is a mosquito-borne RNA virus belonging to the genus *Flavivirus* of the family *Flaviviridae* [2]. The classical clinical picture of ZIKV infection includes fever, exanthema, headache and conjunctivitis. The most common non-congenital, ocular manifestation of ZIKV infection is a self-limiting conjunctivitis. Serious ocular complications have been reported for other arboviruses, such as DENV [3–20], chikungunya virus [21–24], West Nile virus [25–39] and Rift Valley fever virus [9–11, 40] (Table 1). To date, there is no specific ocular lesion that is pathognomonic for ZIKV infection [41–45].

**Table 1.** Non-congenital ocular complications of common Arbovirus infections

Type of virus	Complication
Zika	Uveitis [1, 41, 42]; macular atrophy [46]; chorioretinal scar [47]; chorioretinal macular atrophy [43]
Dengue	Maculopathy or neuropathy [3–8]; macular oedema, retinal detachment, retinal vascular occlusion, choroidal changes, optic disk swelling, optic neuritis and neuroretinitis [3–7, 9–20]
Chikungunya	Optic neuropathy [21]; conjunctivitis, episcleritis, keratitis, panuveitis, multifocal choroiditis, optic neuritis, neuroretinitis, central retinal artery occlusion, panophthalmitis, lagophthalmos and sixth nerve palsy [22–24]
West Nile	Foveal chorioretinal scar, choroidal neovascularization [25]; vitreous haemorrhage secondary to retinal neovascularization, severe ischaemic maculopathy [26]; macular oedema, optic atrophy or retrogeniculate damage; occlusive vasculitis [27–29]; uveitis [30]; vitritis [31]; chorioretinitis [31–35]; multifocal choroiditis [36]; chorioretinal lesions [37]; and optic neuritis [38, 39]
Rift Valley fever	Macular or paramacular scarring, retinal vascular occlusion or optic atrophy [9–11, 40]

Non-congenital ocular complications are infrequent, but serious, consequences of ZIKV and other arboviral infections. The complications may appear at the end of the acute febrile illness, but more commonly occur within 2 weeks to 1 month after the onset of symptoms. There is no evidence to suggest that serious ocular complications correlate with the severity of the acute febrile illness. One study, however, found that the white cell count and serum albumin are significant predictors of ocular complications of DENV [46].

Serological testing for arboviral diseases should be performed in all patients with ocular complications and a recent history of acute febrile exanthematous infection, who live, or have travelled to, endemic regions. The presence of IgM to ZIKV strongly suggests that the ocular manifestation is associated with this virus. A causative aetiology, however, can only be established by documenting the presence of the virus in body fluids, either by cell culture or by PCR. It should be noted that other viruses, such as herpes simplex virus and human immunodeficiency virus, can also cause retinal damage and optic neuritis. Furthermore, as in the cases presented here, the diagnosis is complicated by cross-reactivity among flaviviruses, and by the co-circulation of arboviruses.

Most patients with ocular complications of arboviral infections recover completely. Nevertheless, physicians should be aware that a small percentage of patients have permanent damage with long-life visual impairment.

There is no specific or established treatment for optic neuritis caused by any arboviral infection. Systemic steroids may be used to reduce inflammation and resulting ischaemia. Corticosteroids have been used in combination with acyclovir to treat chikungunya-associated optic neuritis, but efficacy has not been proven [47].
